# Application of machine learning in acute upper gastrointestinal bleeding: bibliometric analysis

**DOI:** 10.3389/fmed.2024.1490757

**Published:** 2024-11-18

**Authors:** Qun Li, Guolin Chen, Qiongjie Li, Dongna Guo

**Affiliations:** ^1^School of Data Sciences, Zhejiang University of Finance and Economics, Hangzhou, China; ^2^Department of Emergency, First Hospital of Shanxi Medical University, Taiyuan, China

**Keywords:** bibliometric analysis (BA), machine learning, AUGIB, research trends, CiteSpace

## Abstract

**Background:**

In the past decade, the application of machine learning (ML) in the clinical management of acute upper gastrointestinal bleeding (AUGIB) has received much attention and has become a hot research topic. However, no scientometric report has systematically summarized and outlined the research progress in this field.

**Objective:**

This study aims to utilize bibliometric analysis methods to delve into the applications of machine learning in AUGIB and the collaborative network behind it over the past decade. Through a thorough analysis of relevant literature, we uncover the research trends and collaboration patterns in this field, which can provide valuable references and insights for further in-depth exploration in the same field.

**Methods:**

Using the Web of Science (WOS) as the data source, this study explores academic development in a specific field from December 2013 to December 2023. The search strategy included terms related to “Machine Learning” and “Acute Upper Gastrointestinal Bleeding”. Only original articles in English focusing on ML in AUGIB were included. The analysis of downloaded literature with Citespace software, including keyword co-occurrence, author collaboration networks, and citation relationship networks, reveals academic dynamics, research hotspots, and collaboration trends.

**Results:**

After sorting and compiling, we have collected 73 academic papers written by 217 authors from 133 institutions in 29 countries worldwide. Among them, China and AM J GASTROENTEROL have made significant contributions in this field, providing many high-quality research achievements. The study found that these papers mainly focus on three core research hotspots: deepening clinical consensus, precise analysis of medical images, and optimization of data integration and decision support systems.

**Conclusions:**

This study summarizes the latest advancements in the application of machine learning to AUGIB research. Through bibliometric analysis and network visualization, it reveals emerging trends, origins, leading institutions, and hot topics in this field. While this area has already demonstrated significant potential in medical artificial intelligence, our findings will provide valuable insights for future research directions and clinical practices.

## 1 Introduction

Acute upper gastrointestinal bleeding (AUGIB) refers to the acute bleeding caused by lesions in the digestive tract above the duodenal suspensory ligament, mainly including the esophagus, stomach, duodenum, bile duct, pancreatic duct, etc. The causes of bleeding, it is divided into two major categories: non-varice upper gastrointestinal bleeding (NVUGIB) and variceal upper gastrointestinal bleeding (VUGIB) (1). Most cases in clinical practice are non-varices upper gastrointestinal bleeding, and the most common causes include gastric and duodenal peptic ulcer, gastric and duodenal erosion, cardiac mucosa tear, and malignant tumors of the upper digestive tract (2). Mild cases may be asymptomatic, and the clinical manifestations are mostly hematemesis, melena, bloody stool, and atypical symptoms such as dizziness, fatigue, and syncope (3, 4). The annual incidence of upper gastrointestinal bleeding in the United States is (48 − 160)/100, 000 people (5), and the annual incidence in Europe is (19.4 − 57)/100, 000 people (6, 7). In China, the annual incidence of AUGIB is (50 − 150)/100, 000 people, with a mortality rate of 2.5–10% and a recurrence rate of 11.9%. More than 300,000 people are hospitalized in the United States each year due to NVUGIB, with costs of up to 2 billion US dollars (8, 9).

Despite advancements in treatment methods, the mortality rate of AUGIB remains high, and the increase in critically ill patients has imposed a heavy burden on the healthcare system (10). Early classification and prognosis assessment are particularly crucial for high-risk patients, allowing for customized and precise treatment plans that emphasize emergency interventions and intensive care while reducing treatment intensity for low-risk patients and optimizing resource allocation. Medical organizations strongly recommend risk stratification for managing AUGIB patients (11, 12). Various scoring models, such as the Glasgow-Blatchford Score (GBS), modified Glasgow-Blatchford Score (mGBS), Pre-endoscopic Rockall Score (PERS), and AIMS65, can guide patient classification, but their effectiveness varies (13). Therefore, there is a need to explore more accurate and rapid diagnostic methods and pay attention to personalized treatment strategies for specific patient populations. Machine learning has enhanced the accuracy of clinical risk stratification by identifying key patterns from medical data (14), thereby conserving medical resources and costs. In the field of cancer classification and diagnosis, ML has made significant research advancements.

To comprehensively grasp the current application status, development trends, and potential challenges of ML in the management of AUGIB, this article utilizes CiteSpace software to conduct a systematic literature visualization analysis. Bibliometrics integrates mathematical and statistical methods to conduct both qualitative and quantitative analyses of literature. It provides insights into the knowledge structure of specific research domains and helps identify emerging trends. By constructing a knowledge map, we have pinpointed current research hotspots as well as potential future research directions and challenges. This study not only provides medical researchers with references and new perspectives but also aids them in effectively applying ML algorithms to facilitate timely identification and appropriate treatment of high-risk patients.

This paper is structured as follows. Section 2 introduces the present the methods used for data collection and analysis. In Section 3, we discuss the results that illustrate key findings related to research trends and collaboration. Section 4 presents conclude with a discussion of these findings and their implications for future research directions. Finally, some conclusions are included in Section 5.

## 2 Materials and methods

### 2.1 Data sources and search strategy

The data in this article are derived from English publications in Web of Science (WOS) over the past decade, from January 1, 2013, to December 31, 2023. In collecting these data, a well-conceived search strategy was employed, which incorporated the keywords to ensure the comprehensiveness and accuracy of the search results.

The search strategy incorporated a comprehensive set of keywords related to “acute upper gastrointestinal bleeding” and “machine learning”, ensuring that all pertinent articles were captured. The following keywords were used to find relevant publications: (((TS=(stomach OR antrum OR antral OR pyloric OR pylorus OR gastri* OR epigastr* OR duodenal OR duodenum OR gastroduodenal OR gastroduodenal OR oesoag* OR espag* OR upper GI OR UGI OR upper gastrointestinal)) AND TS=(hemorrhag* OR bleed* OR rebleed* OR rebleed*)) OR TS=(Hemorrhage, Gastrointestinal OR Gastrointestinal Hemorrhages OR Hematochezia OR Hematochezias OR Gastrointestinal Hemorrhage OR Hematemeses OR Hematemesis OR Melenas OR Melena OR Hemorrhage, Peptic Ulcer OR Peptic Ulcer Hemorrhages OR Ulcer Hemorrhage, Peptic OR Peptic Ulcer Hemorrhage)) AND TS=(computer aided OR data learning OR artificial neural network OR digital image OR convolutional neural network OR evolutionary algorithms OR feature learning OR reinforcement learning OR big data OR image segmentation OR hybrid intelligent system OR recurrent neural network OR natural language processing OR bayesian network OR bayesian learning OR random forest OR multiagent system OR Intelligence, Artificial OR Computational Intelligence OR Intelligence, Computational OR Machine Intelligence OR Intelligence, Machine OR Computer Reasoning OR Reasoning, Computer OR AI Artificial Intelligence OR Computer Vision Systems OR Computer Vision System OR System, Computer Vision OR Systems, Computer Vision OR Vision System, Computer OR Vision Systems, Computer OR Knowledge Acquisition Computer OR Acquisition, Knowledge Computer OR Knowledge Representation Computer OR Knowledge Representations Computer OR Representation, Knowledge Computer OR Artificial Intelligence OR Learning, Machine OR Transfer Learning OR Learning, Transfer OR Machine Learning OR Learning, Deep OR Hierarchical Learning OR Learning, Hierarchical OR Deep Learning OR Learning, Supervised Machine OR Machine Learning, Supervised OR Semi-supervised Learning OR Learning, Semi-supervised OR Semi supervised Learning OR Inductive Machine Learning OR Learning, Inductive Machine OR Machine Learning, Inductive OR Active Machine Learning OR Learning, Active Machine OR Machine Learning, Active OR Machine Learning with a Teacher OR Learning from Labeled Data OR Supervised Machine Learning OR Learning, Unsupervised Machine OR Machine Learning, Unsupervised OR Unsupervised Machine Learning OR Analysis, Sentiment OR Sentiment Analyses OR Opinion Mining OR Mining, Opinion OR Sentiment Classification OR Classification, Sentiment OR Sentiment Classifications OR Sentiment Analysis).

### 2.2 Inclusion and exclusion criteria

The inclusion criteria were as follows: (1) articles must involve the application of machine learning in the management of AUGIB; (2) studies must be related to the diagnosis, treatment, or prognosis of AUGIB; (3) articles must be original research published in peer-reviewed journals; (4) articles must be written in English. Exclusion criteria included review papers, conference papers, data articles, and other non-original research types.

### 2.3 Literature screening process

**Figure 1:**
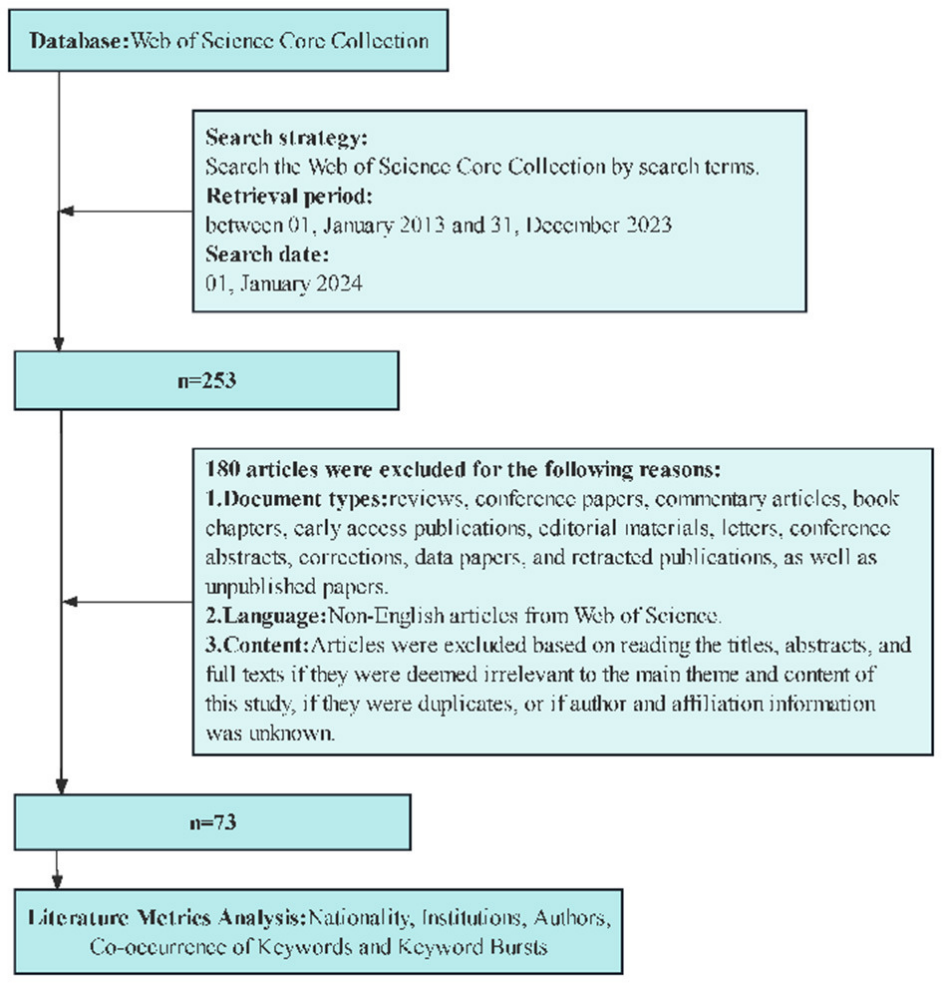
Flowchart of bibliometrics research.

Following the PRISMA strategy, 253 articles were identified. All the literature was screened and checked separately by two researchers to ensure that all the papers used were relevant to the study topic. In the case of a dispute, we consulted a third investigator. After a thorough examination, 73 were included. The English-language articles were exported in full-text format and named ~download_**.txt~ containing information such as authors, affiliations, titles, journal names, abstracts, and keywords. Figure 1 presents a summary of the entire process.

### 2.4 Data analysis

Using CiteSpace (version 6.2.R6), a visual analysis was conducted on the final set of 73 included documents to construct co-authorship networks encompassing countries, institutions, and authors, while also generating knowledge maps for keyword co-occurrence, clustering, and burst detection. The top 50 frequencies were selected, and a suitable K value was determined for each time slice in different projects to optimize network visualization. Pruning techniques, including Pathfinder, slicing, and merging, were applied to refine knowledge maps. CiteSpace visually represents research trends through nodes, links, and colors. Nodes represent entities like authors, institutions, and countries, with sizes reflecting publication count and influence. Link thickness indicates collaboration strength and circular rings around nodes depict citation years and frequencies. In keyword clustering maps, different colors represent clustering areas, with label IDs indicating their order. Clusters with smaller IDs contain more keywords. Significant clustering is indicated by a Q value >0.3, and cluster homogeneity is reflected by a S value >0.7. Nodes with centrality ≥0.1 are considered highly central (15).

## 3 Results

### 3.1 Research status of ML in AUGIB

**Figure 2:**
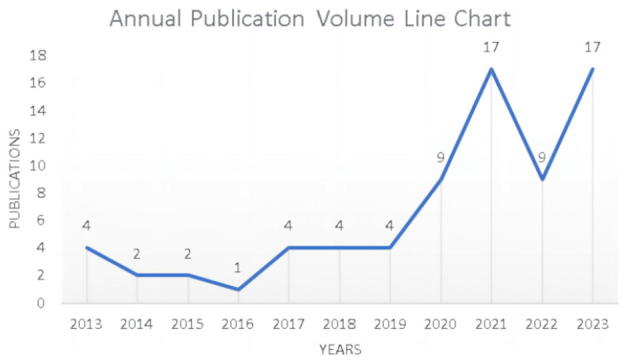
Publication output over time in 2013–2023.

The annual publication trends in the research field provide a visual snapshot of evolving research focuses across various time periods. A review of the research conducted on ML in AUGIB over the past decade indicates a significant surge in the number of publications in the past 4 years. Notably, the peak of this trend emerged in 2021 and 2023, with a total of 34 publications between these 2 years. Figure 2 illustrates the annual publication volume of studies exploring the application of ML in AUGIB.

**Figure 3:**
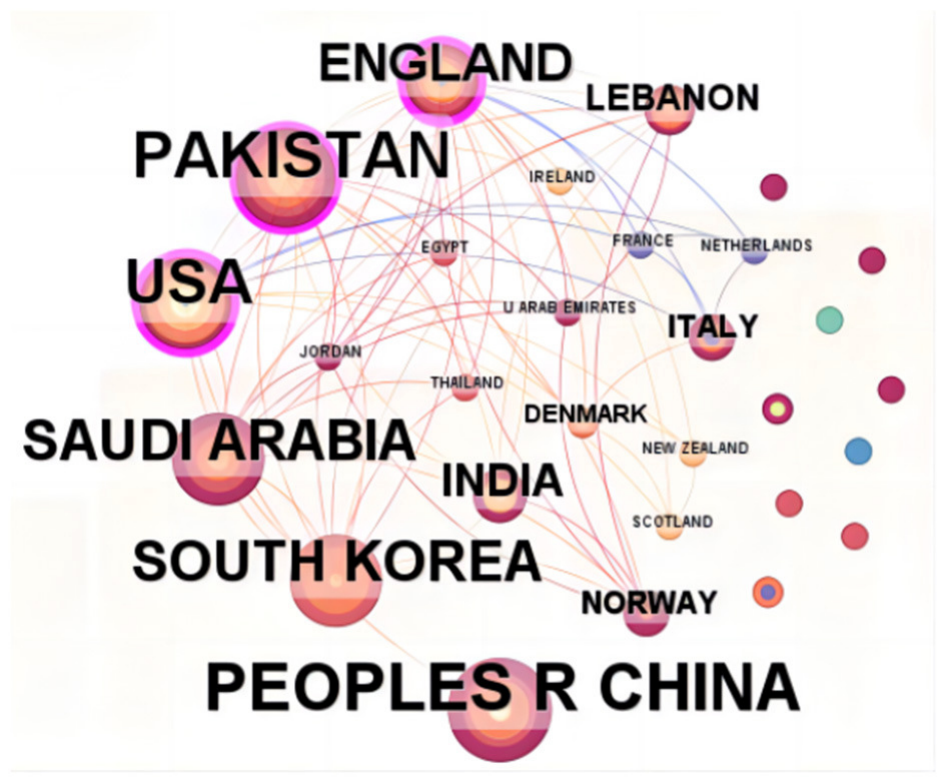
The collaborative network of countries that contributed to the application of ML in AUGIB, 2013–2023.

The research on the application of ML in AUGIB comes from 29 countries/regions, and Table 1 lists the countries that have published more than one study. Among them, the top three countries, ranked by the number of publications, are China (20 papers), Pakistan (17 papers), and the United States (16 papers). Figure 3 illustrates the co-authorship network among countries. Notably, England and Pakistan have stronger ties with other countries, evidenced by their centrality values of 0.65 and 0.37, respectively, indicating well-established collaboration chains.

**Table 1:** Top 10 countries in terms of number of publications.

**Rank**	**Country**	**Publications (*n*)**	**Proportion (%)**	**Centrality**
1	China	20	16.13%	0
2	Pakistan	17	13.71%	0.37
3	USA	16	12.90%	0.1
4	South Korea	12	9.68%	0
5	Saudi Arabia	12	9.68%	0.04
6	England	9	7.26%	0.65
7	India	6	4.84%	0
8	Lebanon	4	3.23%	0.01
9	Norway	3	2.42%	0
10	Italy	3	2.42%	0.03

A total of 133 institutions have published research articles exploring the application of ML in AUGIB, with the top five institutions listed in Table 2. These publishing institutions are primarily composed of higher education or research organizations, including NITEC University (10 papers), COMSATS University Islamabad (CUI) (seven papers), Wuhan University (four papers), University Ha'il (four papers), and Prince Sattam Bin Abdulaziz University (four papers).

**Table 2:** Top five institutions in terms of number of publications.

**Rank**	**Institution**	**Publications (*n*)**	**Proportion (%)**	**Centrality**
1	NITEC University	10	5.59%	0.07
2	COMSATS University Islamabad	7	3.91%	0.05
3	Wuhan University	4	2.23%	0
4	University Ha'il	4	2.23%	0.02
5	Prince Sattam Bin Abdulaziz University	4	2.23%	0.08

**Figure 4:**
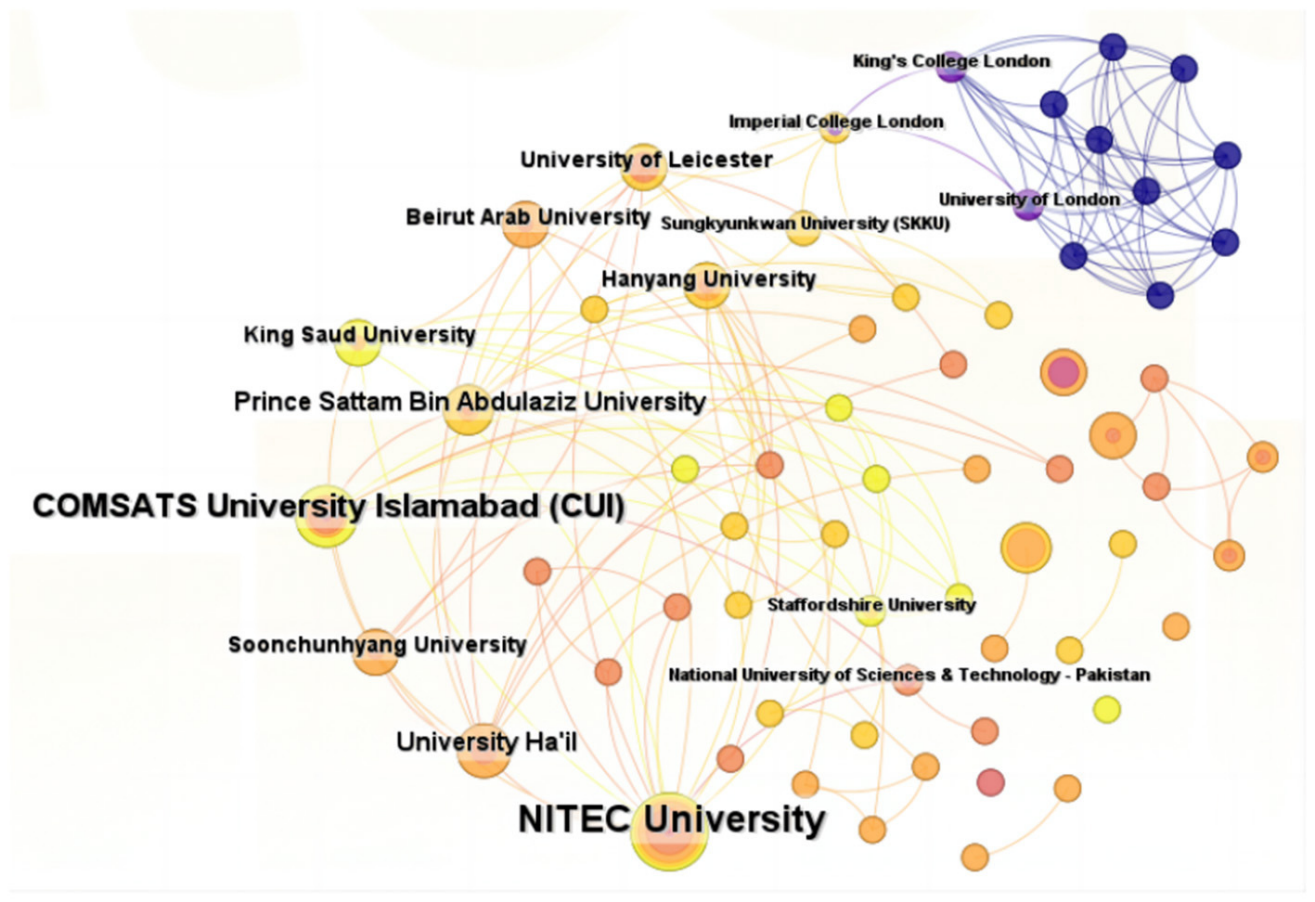
The collaborative network of institutions that contributed to applying ML in AUGIB, 2013–2023.

Figure 4 depicts a robust network of collaborative relationships among these institutions. Notably, the majority of the top ten institutions ranked by publication volume hail from Ukraine, Pakistan, Saudi Arabia, and China, indicating extensive research on the utilization of ML in AUGIB in these countries. However, the size of the labeled nodes in the visualization does not necessarily reflect the number of connecting links, suggesting that institutions with high research output may lack strong ties with others. For instance, despite Wuhan University's significant publication output, it only shows two collaboration links, indicating a preference for domestic collaborations among Chinese institutions in applying ML to AUGIB.

**Figure 5:**
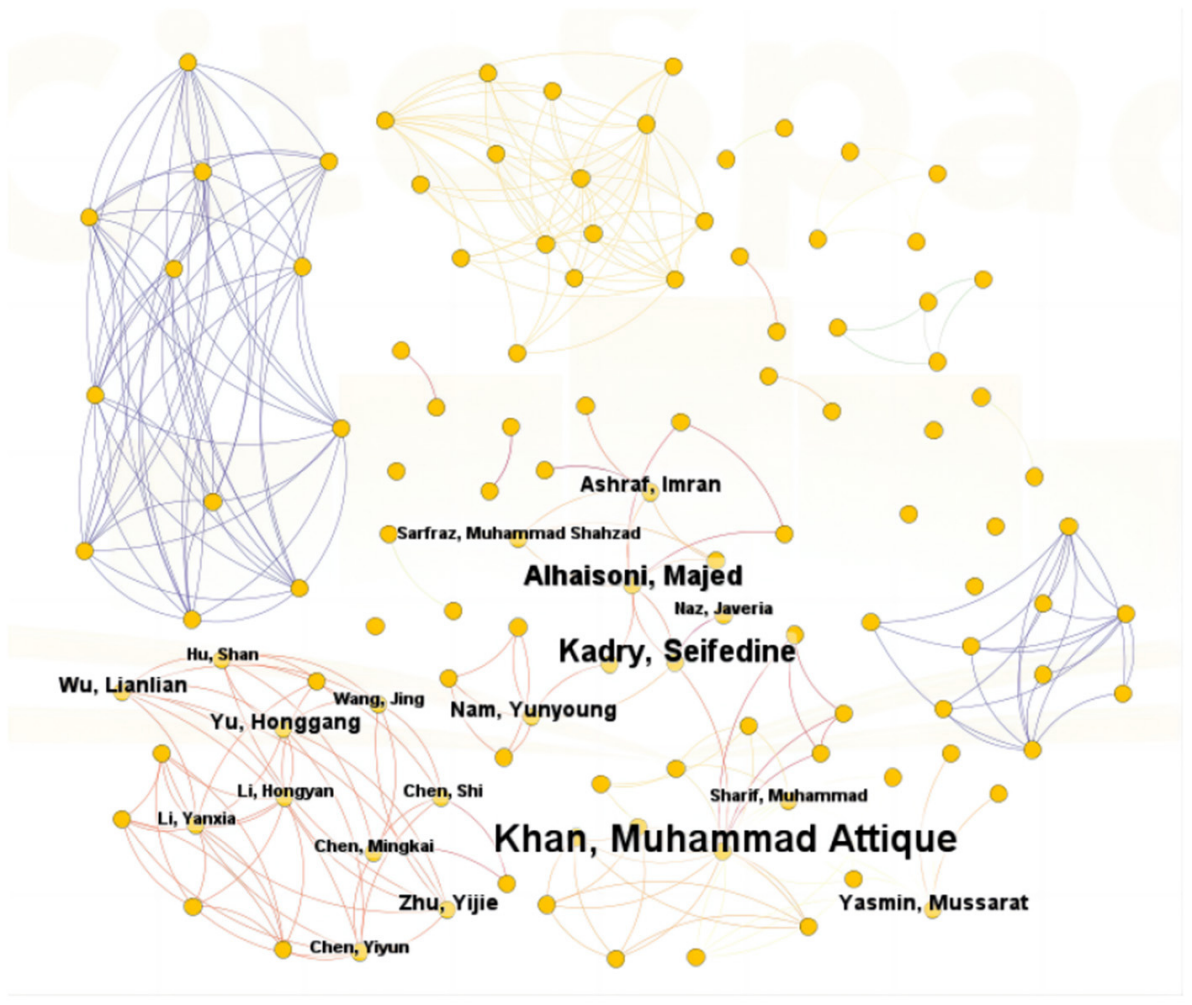
The co-authorship network that contributed tothe application of ML in research on AUGIB, 2013–2023.

A total of 217 authors have researched the application of ML in AUGIB. Table 3 summarizes the publication volume of core authors in the WOS literature. Derek J. de Solla Price, a renowned scientist, and historian of science, posited in his groundbreaking work “Little Science, Big Science” that in any given field (16), approximately half of the total papers are authored by a group of highly productive authors, with the number of these authors roughly equivalent to the square root of the total number of authors contributing to that field. Based on Price's Law (17), if the number of papers authored by the most productive individual in a specific domain is *n*_max_, then $m=0.749*\sqrt{n_{\max}}$. In the context of ML in AUGIB, authors who have published more than m papers are considered primary contributors to this study. Notably, in the WOS dataset, m approximates 2.5, indicating that authors with more than two publications are recognized as core authors. Our analysis identified 22 core authors, with Khan, Muhammad Attique; Kadry, Seifedine; and Alhaisoni, Majed ranking among the top three in terms of publication volume. These authors have significantly influenced the development of this field. Figure 5 visualizes the collaboration relationships among WOS literature authors, highlighting nodes that appear more than twice. Overall, authors studying the application of ML in AUGIB within the WOS literature demonstrate close collaborative ties, forming distinct cooperation circles. These tight-knit scholarly connections are crucial for the in-depth exploration of this domain.

**Table 3:** Top nine authors and co-cited authors that published papers on the application of ML in AUGIB, 2013–2023.

**Rank**	**Author**	**Country**	**Publications (** ***n*** **)**	**Proportion (%)**	**Citation volume**	**Co-cited author**	**Country**	**Citation frequencies**	**Centrality**
1	Khan, Muhammad Attique	Pakistan	11	7.43%	15	Khan MA	Pakistan	15	0.08
2	Kadry, Seifedine	Norway	6	4.05%	0	Anonymous	USA	13	0.5
3	Alhaisoni, Majed Saudi	Arabia	5	3.38%	0	Liaqat A	Pakistan	10	0.07
4	Yasmin, Mussarat	Pakistan	3	2.03%	0	Siegel RL	USA	10	0.01
5	Nam, Yunyoung	South Korea	3	2.03%	0	Li BP	China	9	0.05
6	Ashraf, Imran	South Korea	3	2.03%	0	Laine L	USA	8	0.09
7	Zhu, Yijie	China	3	2.03%	0	Pogorelov K	Norway	7	0
8	Wu, Lianlian	China	3	2.03%	3	Fu YA	China	7	0.1
9	Yu, Honggang	China	3	2.03%	0	Aoki T	Japan	6	0.06

An analysis of the WOS literature on AUGIB reveals that 266 international journals have contributed citations, with Table 4 highlighting the top 10 journals that exhibit the highest citation frequencies, along with their respective citation counts and impact factors. Notably, these 10 journals collectively account for 21.44% of all citations, encompassing 218 papers in total. Among them, “Am J Gastroenterol” emerges as the most cited international journal, boasting 312 articles that account for 6.74% of citations and an impact factor of 9.8. Other prominent journals include “Gastrointest Endosc”, “GUT”, and “Endoscopy”, which also exhibit significant citation counts. “Am J Gastroenterol” firmly establishes itself as a leading clinical journal specializing in upper gastrointestinal issues, providing clinicians with practical and specialized guidance in managing various conditions. Furthermore, the top 10 most-cited journals, including “Lancet”, “New Engl J Med”, and “Gastroenterology”, all originating from the United States, demonstrate impressive impact factors in 2023, indicating significant contributions to AUGIB research outcomes.

**Table 4:** Top 10 cited journals that published papers on the application of ML in AUGIB, 2013–2023.

**Rank**	**Journal**	**Country**	**Referenced count (** ***n*** **)**	**Proportion (%)**	**IF for 2023**	**JAR**
1	Am J Gastroenterol	USA	32	3.15%	9.8	Q1
2	Gastrointest Endosc	USA	31	3.05%	7.7	Q1
3	Gastroenterology	USA	27	2.65%	29.4	Q1
4	Gut	England	21	2.06%	24.5	Q1
5	IEEE Access	USA	19	1.87%	3.9	Q2
6	Lancet	USA	19	1.87%	168.9	Q1
7	CA-Cancer J Clin	USA	18	1.77%	254.7	Q1
8	Endoscopy	Germany	18	1.77%	9.3	Q1
9	Digest Dis Sci	USA	17	1.67%	3.1	Q2
10	J Med Syst	USA	16	1.57%	5.3	Q2

Highly cited literature typically possesses significant research value, attracting widespread attention and discussion within the academic community. It serves as a crucial reference for scholars in the field. Utilizing WOS literature analysis, we have identified highly cited literature pertaining to the application of ML in AUGIB. This literature offers researchers insight into the primary research directions and current hot topics within this field. Table 5 presents the basic information of these highly cited works.

**Table 5:** Top five highly cited references that published papers on the application of ML in AUGIB, 2013–2023.

**Title**	**Journal**	**First author**	**Year**	**Citations**	**Centrality**	**Main content**
Automated ulcer and bleeding classification from wce images using multiple features fusion and selection	Journal of Mechanics in Medicine and Biology	Liaqat A	2018	12	0.07	A principal component analysis (PCA) and correlation coefficient-based feature selection approach is proposed, which is classified by multi-class support vector machine (M-SVM). The proposed method is evaluated on personally collected images of three different classes including ulcer, bleeding and healthy.
Stomach deformities recognition using rank-based deep features selection	Journal of Medical Systems	Khan MA	2019	10	0.04	In this article, a novel computerized automated method is proposed for the classification of abdominal infections of gastrointestinal track from WCE images. Three core steps of the suggested system belong to the category of segmentation, deep features extraction and fusion followed by robust features selection.
Classification of gastrointestinal diseases of stomach from WCE using improved saliency-based method and discriminant features selection	Multimedia Tools and Applications	Khan MA	2019	10	0.06	In this research, a new computer-based diagnosis method is proposed for the detection and classification of gastrointestinal diseases from WCE images.
Validation of a machine learning model that outperforms clinical risk scoring systems for upper gastrointestinal bleeding	Gastroenterology	Shung DL	2020	8	0.00	We used machine learning to develop a model to calculate the risk of hospital-based intervention or death in patients with UGIB and compared its performance with other scoring systems.
Gastrointestinal diseases segmentation and classification based on duo-deep architectures	Pattern Recognition Letters	Khan MA	2020	8	0.09	In this article, a deep learning-based method is presented for ulcer detection and gastrointestinal diseases (ulcer, polyp, bleeding) classification. Modified mask Recurrent Convolutional Neural Network (RCNN) based ulcer segmentation is proposed.

### 3.2 Hotspots and development in ML on AUGIB

**Figure 6:**
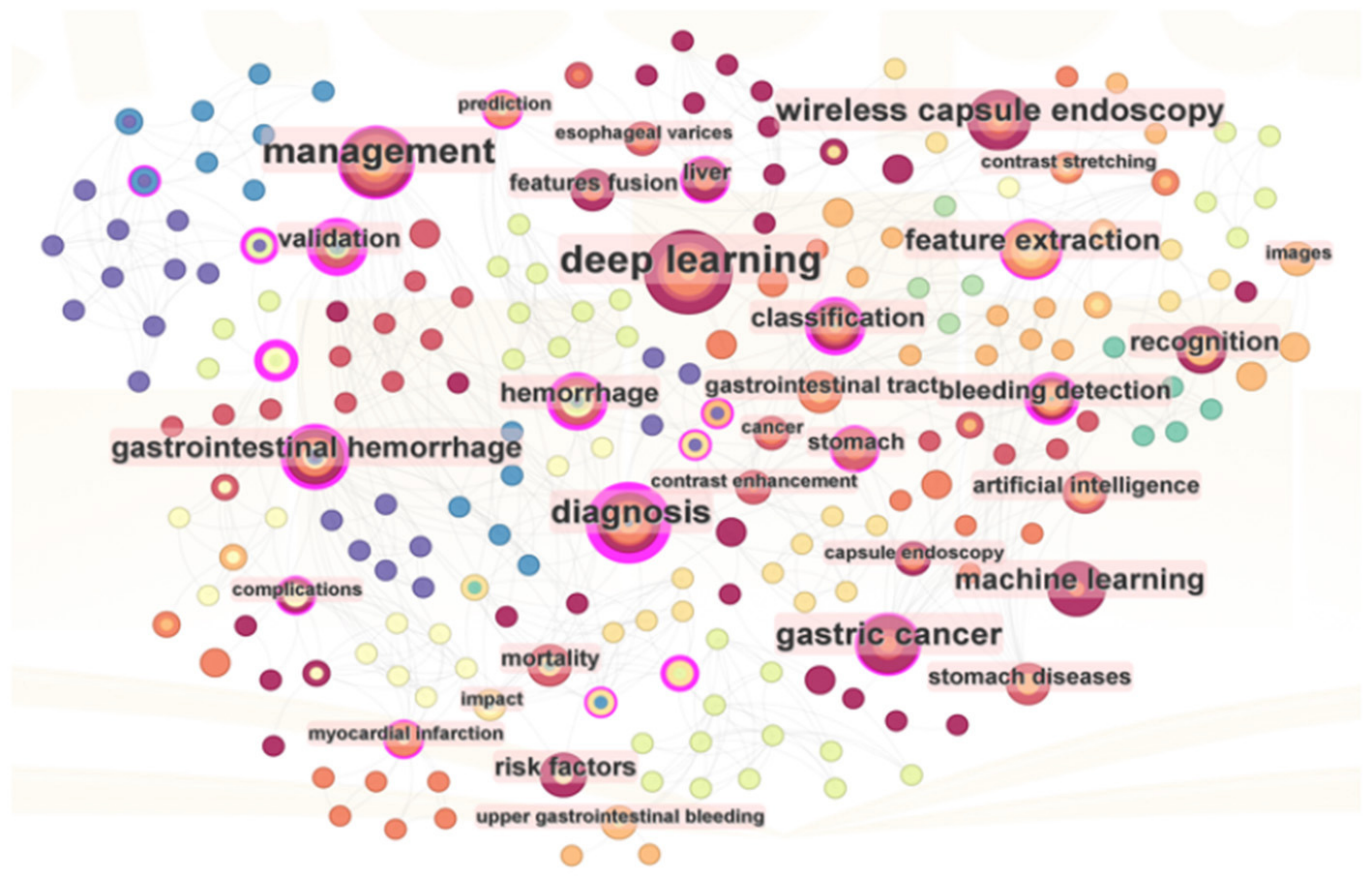
The co-occurrence network of 215 keywords in ML research on AUGIB, 2013–2023.

From a bibliometric perspective, keywords are instrumental in analyzing the knowledge landscape of an academic domain and identifying potential research foci. Citation frequency and intermediary centrality are essential indicators for assessing their importance. The knowledge map in Figure 6 illustrates the keyword network of 215 nodes from literature in WOS, focusing on keywords with frequencies exceeding 2. Furthermore, Table 6 presents a ranking of keywords based on their frequency and centrality.

**Table 6:** Top 10 keywords in frequency and centrality about ML research on AUGIB, 2013–2023.

**Rank**	**Keyword**	**Frequency**	**Keyword**	**Centrality**
1	Deep learning	16	Diagnosis	0.75
2	Management	13	Validation	0.46
3	Diagnosis	10	Gastrointestinal bleeding	0.44
4	Wireless capsule endoscopy	9	Hemorrhage	0.38
5	Gastric cancer	9	Gastrointestinal hemorrhage	0.36
6	Feature extraction	7	Classification	0.34
7	Machine learning	7	Outcom	0.28
8	Gastrointestinal hemorrhage	7	Bleeding detection	0.27
9	Classification	6	Peptic ulcer	0.25
10	Recognition	6	Meta analysis	0.18

Figure 6 highlights “deep learning” as the preeminent keyword. Deep learning, a subset of ML utilizing artificial neural networks, has garnered significant scholarly attention in exploring its application to AUGIB. Notably, keywords with high frequencies often coincide with those ranking high in centrality, indicating a correlation between these two metrics. Centrality encapsulates both research hotspots and pivotal turning points in the field.

**Figure 7:**
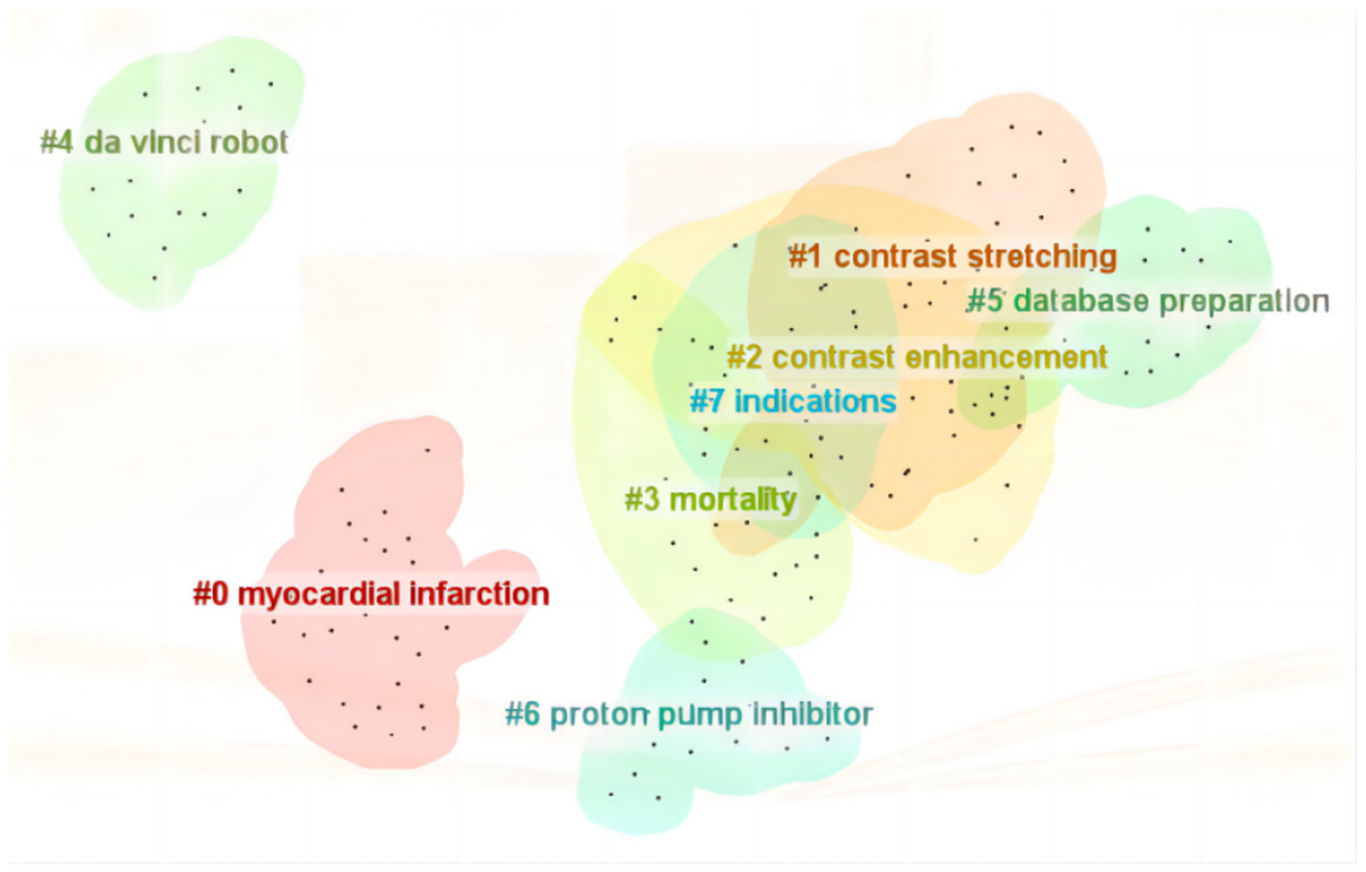
The cluster network diagrams of 215 keywords in ML research on AUGIB, 2013–2023.

Keyword cluster analysis effectively uncovers current research hotspots, with cluster numbers reflecting the descending order of cluster sizes. In the WOS literature, eight primary clusters arise, including myocardial infarction, contrast stretching, contrast enhancement, mortality, da Vinci robot, database preparation, proton pump inhibitor, and indications. The modularity value (Q) of the cluster map stands at 0.8015, significantly surpassing the threshold of 0.3, indicating a distinct community structure. Additionally, the average silhouette coefficient (S) of 0.9294 exceeds the benchmark value of 0.5, further validating the rationality of the clustering results as depicted in Figure 7.

**Figure 8:**
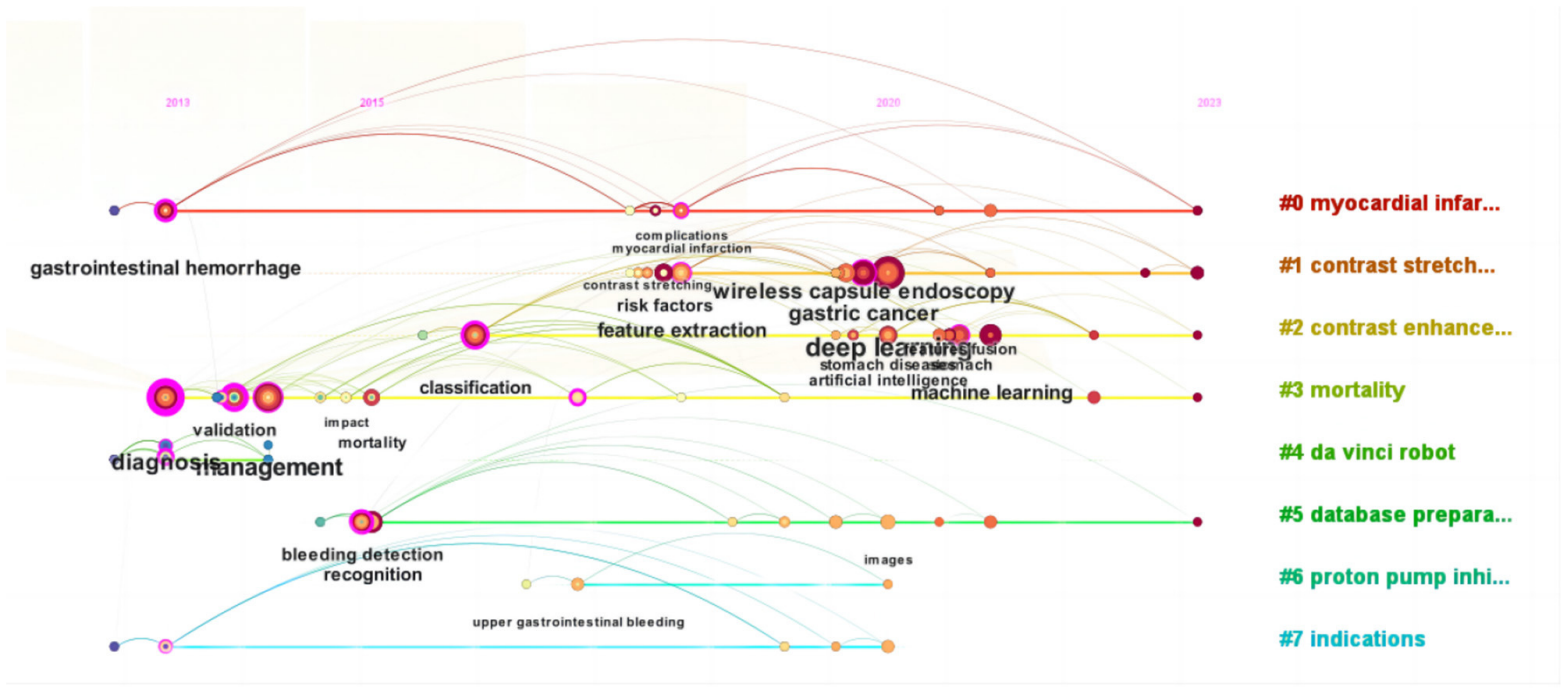
The clustered timeline graph of 215 keywords in ML research on AUGIB, 2013–2023.

By referencing years and clusters, Figure 8 presents a chronological visualization of WOS literature on the application of ML in AUGIB. This visualization comprehensively examines the evolution and advancement of each cluster from 2013 onward, particularly highlighting the emergence of ML and deep learning as prominent research areas post-2020, which has garnered escalating attention and scrutiny through a keyword timeline graph.

**Figure 9:**
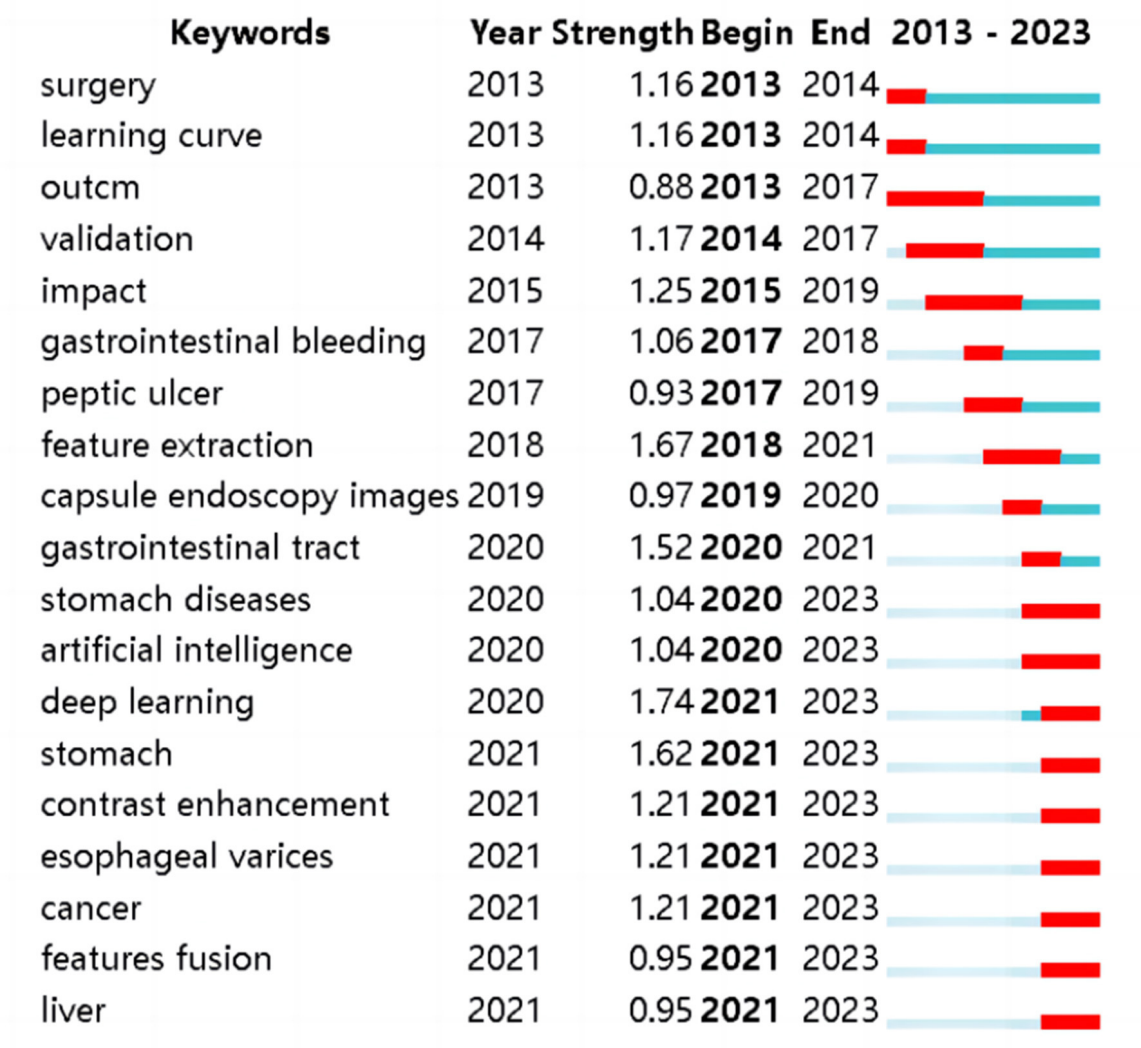
Top 19 keywords with the strongest citation bursts in ML research on AUGIB, 2013–2023.

Based on the high-frequency keyword co-occurrence network, the “Burstness” function was utilized to identify cutting-edge research areas and evolving trends within the field. In the past 4 years, WOS literature has witnessed explosive growth in keywords such as “stomach diseases”, “artificial intelligence”, “deep learning”, “stomach”, “contrast enhancement”, “esophageal varices”, “cancer”, “features fusion”, and “liver”, see Figure 9.

## 4 Discussion

The term “artificial intelligence (AI)”, originally coined in 1956, refers to autonomous learning capabilities tailored for computers (18). A subset of AI, ML, integrates mathematical algorithms to extract insights from unseen data and forecast decisions for predefined tasks. AI has developed rapidly and has applications in several disciplines, among which machine learning is a rapidly evolving technology frontier (19). AUGIB, a common clinical challenge affecting 25–35 hospitalizations per 100,000 annually, varies in severity, and predicting its clinical course remains challenging. As a result, most patients with suspected AUGIB receive inpatient and endoscopic evaluations within 24 h, which strains hospital resources, causes patient discomfort, and increases healthcare costs (20). ML algorithms provide opportunities for accurate prognostic prediction, risk stratification, and optimal clinical management.

This study employed bibliometrics to delve into the research dynamics and future trends of ML in AUGIB between 2013 and 2023. Significant advancements in the field have been observed since 2020, peaking in 2021 and 2023, with predicted continued growth in the future. China boasts the most extensive research output in this field, and international collaboration is also highly active, with Pakistan and the United Kingdom occupying pivotal positions in the global collaboration network. Key institutions such as NITEC University, COMSATS University Islamabad, and Wuhan University have emerged as significant contributors and influencers in this field, underscoring the crucial role of educational and research institutions in driving medical science advancements. Authors like Khan, Muhammad Attique, and Kadry, Seifedine have exerted profound impacts on the application of ML in AUGIB. While Chinese scholars have a high volume of publications, their average citation rate remains relatively low, indicating a need to enhance the impact of their work. Additionally, international collaborations among Chinese and foreign scholars remain insufficient compared to those among international institutions. Highly cited journals such as Am J Gastroenterology and Gastrointest Endosc reflect the US's preeminent contributions in this domain, while top-cited articles primarily focus on classification algorithms and feature selection, exerting significant global influence on ML practices in AUGIB management.

The findings of this study suggest that ML holds considerable promise in managing AUGIB, particularly in risk stratification, prognosis prediction, and image analysis. However, it is essential to recognize that VUGIB and NVUGIB exhibit fundamentally distinct clinical characteristics. The VUGIB is typically associated with portal hypertension and necessitates specific interventions such as band ligation or transjugular intrahepatic portosystemic shunt (TIPS). In contrast, NVUGIB is often attributed to peptic ulcers or erosive conditions, which require different management strategies and prognostic considerations. This differentiation is critical for comprehending the tailored applications of ML across various clinical scenarios. A key limitation in the current study is the lack of sufficiently large datasets specifically focused on either VUGIB or NVUGIB. Most studies have aggregated data without clearly distinguishing between the two conditions, leading to a combined approach in our bibliometric analysis. Data sharing across institutions and the establishment of larger, more diverse datasets are critical for the development of specific, robust ML models tailored to each type of AUGIB. Furthermore, improving the explainability of ML models remains a critical challenge across both types of bleeding, as clinicians need to understand the underlying factors driving predictions to effectively integrate these tools into practice.

Through keyword analysis and cluster analysis, this study revealed the main research hotspots of ML application in AUGIB, focusing on clinical consensus topics such as “management”, “diagnosis”, and “proton pump inhibitor”. In addition to clinical management, this study also emphasizes indicators such as clinical diagnosis, drug therapy, and mortality prediction, showing a strong interest in the actual outcomes of treatment.

The European Society of Gastrointestinal Endoscopy, Asia-Pacific Working Group, and international consensus groups advise risk stratification for patients with UGIB (21), which is essential for predicting disease severity, clinical need, and prognosis. Existing risk scoring systems such as Rockall Score (RS), Glasgow-Blatchford Score (GBS), AIMS65 Score, and ABC Score, vary in assessing patients, especially in NVUGIB. Given that VUGIB patients often present with underlying liver disease, alternative scoring systems such as the Child-Pugh classification, the Model for End-Stage Liver Disease (MELD) score, and the MELD-Na score are frequently used instead of the standard AUGIB scoring systems. In 2003, Barkun et al. published consensus recommendations for NVUGIB treatment, sparking further research on AUGIB mechanisms and symptom management (22). In 2015, the Emergency Physicians Branch of the Chinese Medical Association issued an expert consensus, emphasizing the importance of risk stratification in AUGIB to help early detection of life-threatening bleeding and strengthen intervention (13). Furthermore, in 2022, the Chinese Society of Hepatology of the Chinese Medical Association specifically issued guidelines for the management of VUGIB in patients with cirrhosis (30). Traditional risk grading and scoring systems standardized emergency treatment. Since 2020, ML algorithms have been increasingly integrated into clinicians' risk assessment and decision-making.

Across diverse predictions, ML models demonstrated remarkable accuracy in predicting outcomes among patients with AUGIB. A comprehensive evaluation encompassing multiple models yielded a median AUC score of 0.82, ranging from 0.65 to 0.95. Notably, in specific studies led by researchers such as Shung, Dennis L et al., the predictive capabilities of these models surpassed those of traditional clinical risk scores. In one study, a gradient-boosted ML model achieved an AUC of 0.90, significantly outperforming clinical risk scores like the admission Rockall, AIMS65, and Glasgow-Blatchford scores (23). This superior performance facilitated the identification of low-risk patients suitable for outpatient management, a crucial aspect in resource allocation and patient care. Another study compared ML models to the GBS in predicting outcomes among patients with AUGIB. In both internal and external validation cohorts, the ML models demonstrated superior predictive accuracy, with AUCs of 0.88 and 0.90, respectively (24). This robust validation underscores the generalizability and predictive power of ML models in risk stratification for AUGIB patients. Furthermore, a study utilizing electronic patient records developed an interpretable ML model that significantly outperformed the widely used APACHE IVa scoring system in predicting mortality among patients with AUGIB in intensive care units (25). This finding highlights the potential of ML models in enhancing risk prediction and patient management strategies in critical care settings.

This study utilizes keyword and cluster analysis to reveal that apart from clinical consensus, medical imaging applications, particularly those pertaining to “contrast stretching”, “contrast enhancement”, and “deep learning”, have emerged as key research foci, aimed at enhancing image quality and facilitating accurate analysis and diagnosis. ML has been a pivotal force in revolutionizing AUGIB medical image analysis, significantly advancing the field's diagnostic capabilities and treatment strategies. Convolutional neural networks (CNNs) have emerged at the forefront of image analysis techniques, demonstrating remarkable performance in extracting meaningful features from complex medical images.

Deep learning models, particularly those tailored for medical image analysis, have achieved remarkable accuracies in diagnosing endoscopic and other medical images related to AUGIB. These models can swiftly identify bleeding sources, significantly enhancing diagnostic speed and accuracy. This, in turn, enables clinicians to initiate appropriate treatment measures promptly, potentially reducing morbidity and mortality rates associated with AUGIB (26, 27). Collaborative studies involving artificial intelligence and endoscopists have further underscored the diagnostic superiority of ML in gastrointestinal diseases. Comparative studies have demonstrated that AI-based classifiers often outperform even experienced endoscopists in detecting early gastric cancer and other gastrointestinal pathologies (28). These findings highlight the transformative potential of ML in AUGIB management, pointing to a future where AI becomes a standard tool in clinical decision-making.

The application of ML in AUGIB management is not limited to image analysis. It optimizes treatment decisions by leveraging vast medical databases, enabling clinicians to design personalized treatment plans based on the individual needs of patients. The ML model combines “database preparation” and “feature fusion” to integrate disparate feature data, improving performance and robustness, which is critical for multimodal AUGIB-related data. Compared to traditional risk classification systems, ML demonstrates superior data integration and decision support capabilities. It processes complex medical data, including imaging, laboratory results, and patient history, enabling comprehensive analysis (29). ML models can dynamically adapt and improve new data, enhancing adaptability to evolve medical knowledge. Feature learning enables machine learning to discover complex patterns and associations in patient data, going beyond reliance on predefined rules. In addition, personalized predictions tailored to individual patient situations enhance the usefulness of ML in AUGIB management.

However, they also present several limitations. For example, the interpretability of complex models, such as deep learning, remains a significant challenge, which may limit their clinical adoption. Many healthcare professionals require a clear understanding of how these models make decisions, and the lack of transparency can hinder trust and application in clinical settings. Additionally, the majority of current studies have used retrospective data, which may introduce biases and limit the generalizability of the findings to broader patient populations.

## 5 Conclusion

Through bibliometric analysis, it can be inferred that the application of machine learning (ML) in the evaluation and prediction of Acute Upper Gastrointestinal Bleeding (AUGIB) is attracting more and more attention. In the past 3 years, ML has become a hot topic in diagnosis, image analysis, risk assessment, treatment recommendation, prognosis prediction, and other aspects, indicating the future trend. With the development of artificial intelligence technology, multi-center research and information management will push the field forward. We expect to provide more accurate support for AUGIB diagnosis and treatment by improving clinical guidance, developing AI prognostic models, and combining new technologies. Compared with traditional approaches, machine learning classification strategies promise to reduce costs.

However, the study has limitations, such as a single database and missing searches. To ensure the reliability of machine learning, high-quality data needs to be widely collected and rigorously validated. At the same time, model interpretability and patient privacy protection are crucial. In the future, machine learning should be used as an aid to medical decision-making to fully leverage their application value.

## Data Availability

The original contributions presented in the study are included in the article/supplementary material, further inquiries can be directed to the corresponding author.
